# Dr. Charles Clifford Barrows

**Published:** 1916-03

**Authors:** 


**Dr. Charles Clifford Barrows,** N. Y. Univ. 1880, died Jan. 3, aged 58, at Ithaca. He was Prof. of Gynaecology at Cornell and formerly resided in New York City.

